# Statistical reviewing for a medical journal: summary of workshop proceedings

**DOI:** 10.1186/s41073-026-00227-w

**Published:** 2026-06-15

**Authors:** Derrick Bennett, Sam Leary, Paula Dhiman, Jen Lewis, Julie Morris, Gordon Prescott, Andy Vail, Ashma Krishan, Antonia Marsden, Clare Robinson, Jamie Sergeant

**Affiliations:** 1 Clinical Trial Service Unit and Epidemiological Studies Unit (CTSU), Nuffield Department of Population Health (NDPH), Big Data Institute, University of Oxford, Old Road Campus, NuffieldOxford, OX3 7LF UK; 2 Bristol Dental School, University of Bristol, Bristol, UK; 3 Centre for Statistics in Medicine, Nuffield Department of Orthopaedics, Rheumatology and Musculoskeletal Sciences (NDORMS), University of Oxford, Oxford, UK; 4 Sheffield Centre for Health and Related Research (SCHARR), School of Medicine and Population Health, University of Sheffield, Sheffield, UK; 5 Retired Head of Medical Statistics, South Manchester NHS Trust, Manchester, UK; 6 Lancashire Clinical Trials Unit, University of Lancashire, Preston, UK; 7 Centre for Biostatistics, University of Manchester, Manchester, UK; 8 Wolfson Institute of Population Health, Queen Mary University of London, London, UK

**Keywords:** Statistical, Reviewing, General medical journals, Recommendations

## Abstract

The use of statistical methods in medical research has long been a concern and careful review of these methods is required to ensure that published papers are of sufficient statistical quality. However, there is a shortage of statistical reviewers for medical journals and there is a dearth of training for those with little or no experience.

An online workshop on statistical reviewing for medical journals was convened by the Improving Statistical Literacy and Early Career Development streams of the National Institute for Health and Care Research (NIHR) Statistics Group to address this gap.

This article summarizes the key learnings from this workshop and provides some practical advice for those potentially interested in undertaking statistical reviews for a medical journal.

## Introduction

There has been a substantial increase in the use of statistics in medical research and thus in papers published in medical journals. A 2017 review of general medical journals found that there is an increasingly wide range of methods that need to be understood by readers of public health journals [1]. Just over 30 years ago, Altman lamented the “scandal of poor medical research” and relayed instances of researchers using the inappropriate techniques (intentionally or unintentionally), misinterpreting their results or drawing conclusions that were not justified from the results presented [2].

There is a clear need for a statistical review of manuscripts submitted to medical journals, but a recent survey of biomedical journals noted that 34% (36/107) of journal editors rarely or never use a specialized statistical review [3]. This is consistent with an earlier cross-sectional survey that found that with the exception of the largest circulation medical journals (e.g. New England journal of Medicine, Lancet, BMJ) the probability of a formal methodological review of clinical research was low [4].

Traditional peer review involves inviting subject matter experts in the field to assess the originality, validity and importance of a manuscript. Whereas statistical review focuses on the statistical methods and analyses used in the manuscript in order to assess whether they are appropriate, correctly implemented and are transparent and robust.

Statistical rigour is important for ensuring the validity of research findings and poor statistical practices such as inadequate description of methods, errors in inference and conclusions may go unnoticed by traditional peer reviewers [5, 6].

Providing a statistical review for a medical journal can be a rewarding experience, but there is little formal training on how to go about it. This article summarises the presentations and discussions from an interactive online workshop introducing statistical reviewing for medical journals which was jointly organised by the Improving Statistical Literacy Improving statistical literacy | NIHR Statistics Group) and Career development (Career development | NIHR Statistics Group) working groups of the National Institute for Health and Care Research (NIHR) Statistics Group and held on 20 January 2025. All of the co-authors of this report were either speakers, practical facilitators or organisers of the workshop.

The aims of the workshop were to: a) give an overview of why statistical reviewing is important; b) describe how to conduct a statistical review; c) provide specific guidance on how to provide a statistical review for a clinical trial or observational study; d) give practical advice on how to get involved in statistical reviewing for medical journals.

### Target audience, workshop content and format

Invitations to register for this event were sent to statisticians, epidemiologists and quantitative researchers via the NIHR Statistics Group mailing list, the authors’ professional networks, social media, mailing lists and institutions. For logistical reasons we decided to conduct an online workshop as we aimed to reach a wider statistical audience across the UK without participants having to travel to a specific location. Moreover, it would have created a logistical challenge (e.g., room bookings, catering) to conduct an in-person workshop.

In total, 167 people registered to attend the workshop, excluding the contributors. As part of the registration attendees were asked to specify whether their skills and interests were more in clinical trials or observational studies and about their previous statistical reviewing experience.

The workshop was split into two main sessions. The first session included a series of 10-min talks that addressed aims a-c. The short talks were followed by a 20-min question-and-answer session with the speakers. Each of the speakers had had first-hand experience of statistical reviewing for a general medical journal. The second session was a practical statistical reviewing exercise. Participants were sent the appropriate paper (observational study or clinical trial) ten days before the online workshop, and asked to spend approximately one hour reading it, focusing on the abstract, methods and results, noting down their initial thoughts in preparation for the on-line workshop. For the 45-min practical exercise, participants were split into five groups; two groups reviewed the observational study paper [7], and three groups reviewed the clinical trial paper [8]. Groups were split as evenly as possible in terms of previous statistical reviewing experience, and each had allocated facilitators to lead the discussion within the session.

Following the workshop, attendees were invited to complete a feedback form rating various aspects of the webinar, and indicating the extent to which their expectations had been fulfilled.

### Summary of proceedings

The target audience were UK-based statisticians and quantitative researchers from a range of institutions and career stages but with limited experience of statistical reviewing; 69.9% had never conducted a statistical review, 7.2% had written at least one review but under the guidance of a mentor, and 22.9% had written at least one review on their own (N = 166; 1 missing response). The subsequent sections relate to individual short talks addressing overall aims a-c of the workshop.

#### Why be a statistical reviewer?

##### Personal Development

A statistical reviewer is likely to encounter wide-ranging information regarding statistical applications in medicine and have a direct window on research outside their own area of expertise. An unfamiliar statistical approach in a review paper can provide a unique learning opportunity. Before making a considered judgement on the paper, the statistical reviewer may need to look up details of the technique, familiarise themselves with the methodology, and find references to published research studies where this method has been put into practice.

The papers that the statistical reviewer will encounter should have detailed information about different methodological techniques, patient selection, management of various interventions, avoidance of bias and so on. This will add to their own knowledgebase, ultimately giving a better understanding of the practical issues relating to research studies. Of course, being a statistical reviewer adds greatly to the skills that can be listed in a CV. In addition, statistical reviewing is part of “good research citizenship” and can contribute to promotion applications.

##### Promoting good statistical practice

The statistical reviewer has the opportunity to use their statistical expertise to educate the authors of research papers. Incorrect statistical tests can be identified, more optimal analysis may be recommended, or additional statistical analyses can be suggested that would improve the paper [9]. Methodological design flaws may also be a consideration.

##### Wider impact

Statistical reviewers contribute to the improvement of the statistical quality of published papers and have a part to play in the role that published evidence has on the decisions made to introduce newer medicines/techniques into clinical practice. In addition, statistical reviewers may even help to detect fraudulent studies by identifying unusual data or ‘too perfect’ results in submitted papers, and prevent these papers from being published [10].

#### How to review a paper from a statistical viewpoint

It will usually be possible after reading the abstract to determine whether the clinical context, research design and statistical analysis methods are sufficiently familiar to continue. Although statistical reviewing can provide a unique learning opportunity, if after thoroughly investigating the methodology, the reviewer feels that a second opinion may be required, journal editors will appreciate being alerted to this.

##### Approach to the statistical review

It can be helpful to read the whole manuscript, making brief notes of specific points, before beginning to write the review. This will allow the statistical reviewer to form a broad opinion as to which category best describes the overall statistical approach: 1) not misleading, so ‘minor changes’ will be requested; 2) salvageable, requiring ‘major changes’; or 3) there is a fundamental flaw that makes the results unreliable therefore the findings are not worth sharing with the wider scientific community, justifying ‘reject’.

##### Structure of the statistical review

The review itself will usually start with a sentence or brief paragraph summarising the manuscript. This gives reassurance to the editor/author that the reviewer has read and understood the submission. A list of ‘major’ issues typically follows, in order of either importance or appearance in the text. Specific line numbers (if given) or locations (e.g., “Methods, third paragraph, second line”) will avoid ambiguity for editors and authors alike. A similarly structured list of ‘minor’ issues should then conclude the review.

A ‘major’ statistical issue may be any aspect of design, analysis or interpretation that has led to an unsafe conclusion based on the available data. Examples may include unrecognised statistical artefacts or epidemiological fallacies in the design; seriously misleading graphs or failure to use appropriate analyses. Whilst ‘major’ issues are common in submitted manuscripts, it is rarely impossible to salvage a worthwhile presentation and interpretation of the underlying data.

##### Recommendations to editors and authors from the statistical review

The reviewer should always acknowledge aspects of the methodology, (despite reading up on the methods), where they may be out of their depth. The reviewer should tailor comments to the likely expertise of the recipient (e.g. statistician or clinician). Finding positives in the manuscript is important to help authors receive the comments and suggestions. Clear, concise, specific and constructive criticism will help the editors as well as the authors.

#### Specific statistical considerations for an epidemiological paper

##### Research question

The statistical review of epidemiological studies depends to some extent on the study design that is being used to answer the research question. Though there can be differences between study designs, many aspects of a study that need to be reviewed are similar regardless of the design. The research question sets the scene for any research study, informing the statistical reviewer of important information about the study: the PECO (Population, Exposure, Comparator, Outcome) [11] and what the sample is intended to be used for. The research question and the aims of the study are often given in the last paragraph of the introduction section.

##### Methods section

Knowing the research question allows a more efficient review of the methods and their key assumptions. The methods section, which depends on the research question, is perhaps the most important section, and is where most time is spent when statistically reviewing an epidemiological paper. For example, each study design using an observational dataset will need its own bespoke sample size calculation [12] and the key methodological aspects of a prediction model study will differ greatly from a causal inference study. Concerns have been raised around errors by researchers and a lack of ability to appropriately distinguish between causal, predictive or descriptive research [13].

##### Results section

The results section should be scrutinized for results that do not match the methods through unspecified analyses or omitted results. The results section can also be a place to find issues related to p-hacking [14] and data dredging [15]. It is also useful for authors to attempt to perform sensitivity analyses that quantify the potential impact of biases on the validity of their findings [16].

##### Discussion section

Knowing the research question, the methodology and results, will allow for the strengths and limitations of the analysis to be considered. Often authors will acknowledge the strength and limitations of their study in the discussion, where they can also highlight the impact of these on their results. However, if strengths and especially limitations of any study have not been reported, or if there is evidence of “spin” this can be considered as a red flag, as we know the perfect research study does not exist [17].

#### Specific statistical considerations for a clinical trial paper

##### General information

The statistical reviewer should read the clinical trial paper to ensure that it has an informative title, a clear research question, details of trial registration, evidence of ethical approval and a declaration of any potential conflicts of interest.

##### Methods section

Methods should match the published protocol. Reviewers should ensure that inclusion and exclusion criteria are justified, and that both intervention and control are clearly described [18, 19]. Methodological clarity is crucial to enable replication and there should be a reproducible sample size calculation and evidence of an a-priori statistical analysis plan [20]. The statistical reviewer should examine participant selection, randomisation method, allocation procedure and planned statistical analysis for any evidence of inherent bias.

##### Results section

There should be a clear diagram of the participant flow, explanations for ineligible or non-randomised participants and consistency in the number of participants reported throughout. Departures from assumptions, and large attrition or attrition disparity between treatment groups should be acknowledged and justified.

##### Discussion section

The statistical reviewer should expect potential biases of the trial to be discussed alongside strengths and limitations in a fair and balanced way [21].

#### How to get involved in statistical reviewing: reflections on first experiences of statistical reviewing

The final short talk focused on the first experiences of statistical reviewing of an early career researcher (aim d of the workshop). Table 1 briefly summarizes the reflections and the challenges that were faced and how they were overcome.

**Table 1:** Personal reflections on first experiences of statistical reviewing

The opportunity to perform a statistical review arose around two years into my first statistician role. This first statistical review was challenging and involved methods that I hadn’t had direct experience of, including boosted logistic regression, propensity scores and nearest-neighbour matching. Despite some trepidation, this felt like a good learning opportunity, but given my inexperience with the methods I ensured that I had supportive measures in place. These measures were critical to ensuring I was able to provide a thorough and high-quality review To complete the review, I had to spend time reading about the methods I was going to be reviewing in a reasonable amount of detail, including the principles, implications and pitfalls of the analyses, so I made sure that I had the capacity to take it on. Before agreeing to conduct the statistical review, I made sure that there were colleagues around me with experience of these methods, and the time to provide advice if I needed it; importantly having sought permission from the editor to get another pair of eyes on the paper. It was a positive experience, and a great way to develop an understanding of different methods in practice. It became an important contribution to my CV, and gave me perspective about my own knowledge and skills	

#### Practical exercise summary

One hundred and twelve participants had joined for session one of the online workshop, and ninety participants (80%) of these stayed for the practical session, with group sizes ranging from 14 to 20. Within each group, the facilitator gave a brief overview of the paper, then began the discussion by asking participants to highlight positive aspects. Participants were then asked to discuss their views on the overall paper conclusion, in particular whether it followed from the analyses presented. Discussion of concerns or clarifications required for each aspect of the paper such as the abstract, tables and figures filled the remainder of the time, with facilitators providing prompting questions.

At the end of the allotted time all participants reconvened, and each facilitator provided a brief summary of their session. Major flaws of the papers, such as poor readability, lack of clarity about study design including whether trials were pilot or definitive, poor sample size justification, incorrect or unclear justification for statistical analysis, lack of confidence intervals, inappropriate interpretation of p-values and overly optimistic conclusions were highlighted.

#### Feedback from participants

Just over half of the participants (N = 87, 51.8%) completed the online feedback form. 

**Fig. 1:**
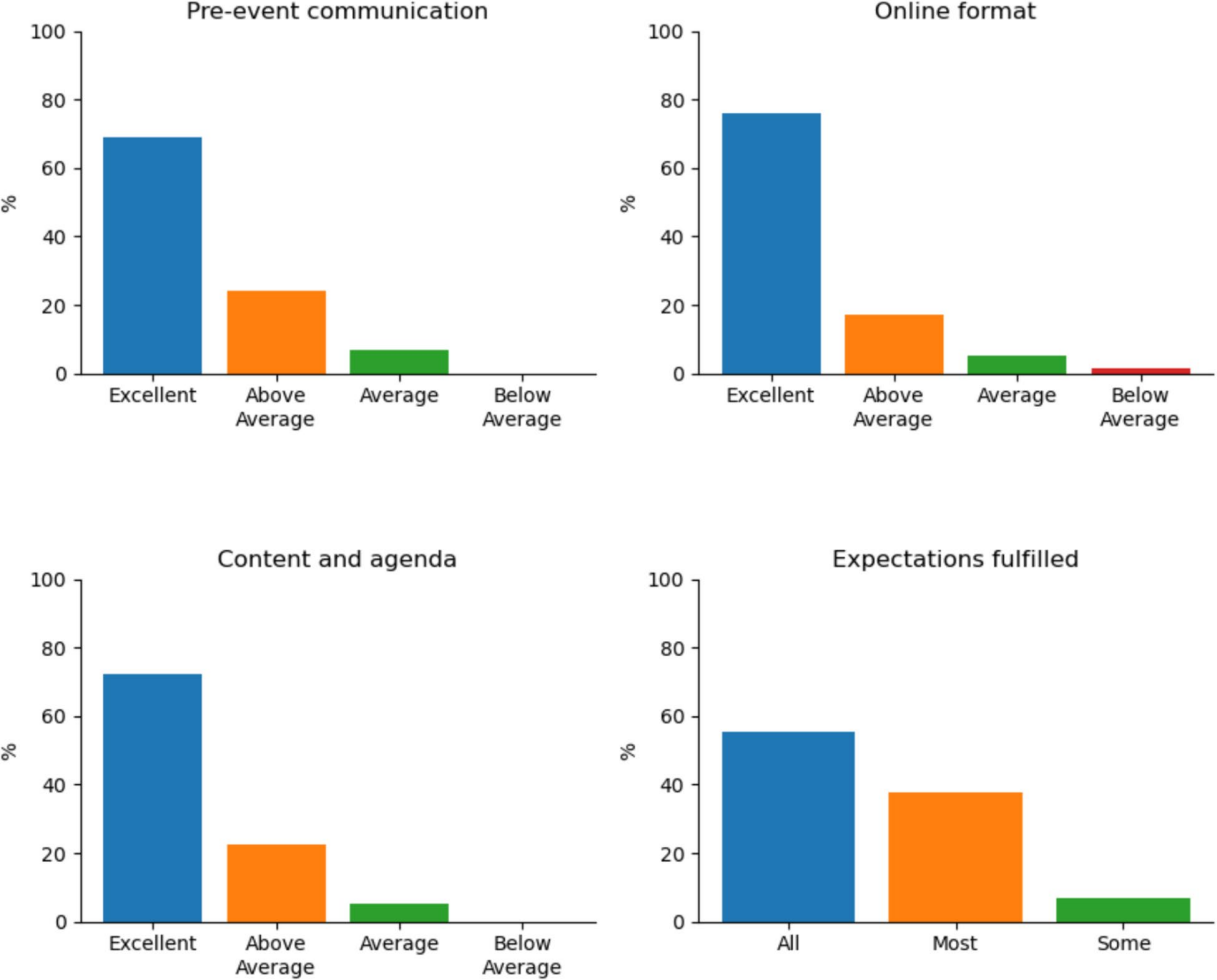
Workshop participants ratings for general questions

Figure 1 summarizes the responses from participants regarding pre-event communication, the online format, the content and agenda, and to what extent expectations were fulfilled. Pre-event communication and online format were rated as excellent or above average by 93%, the content/agenda was rated as excellent or above average by 95%. All or most expectations were fulfilled for 93% of the participants.

**Fig. 2:**
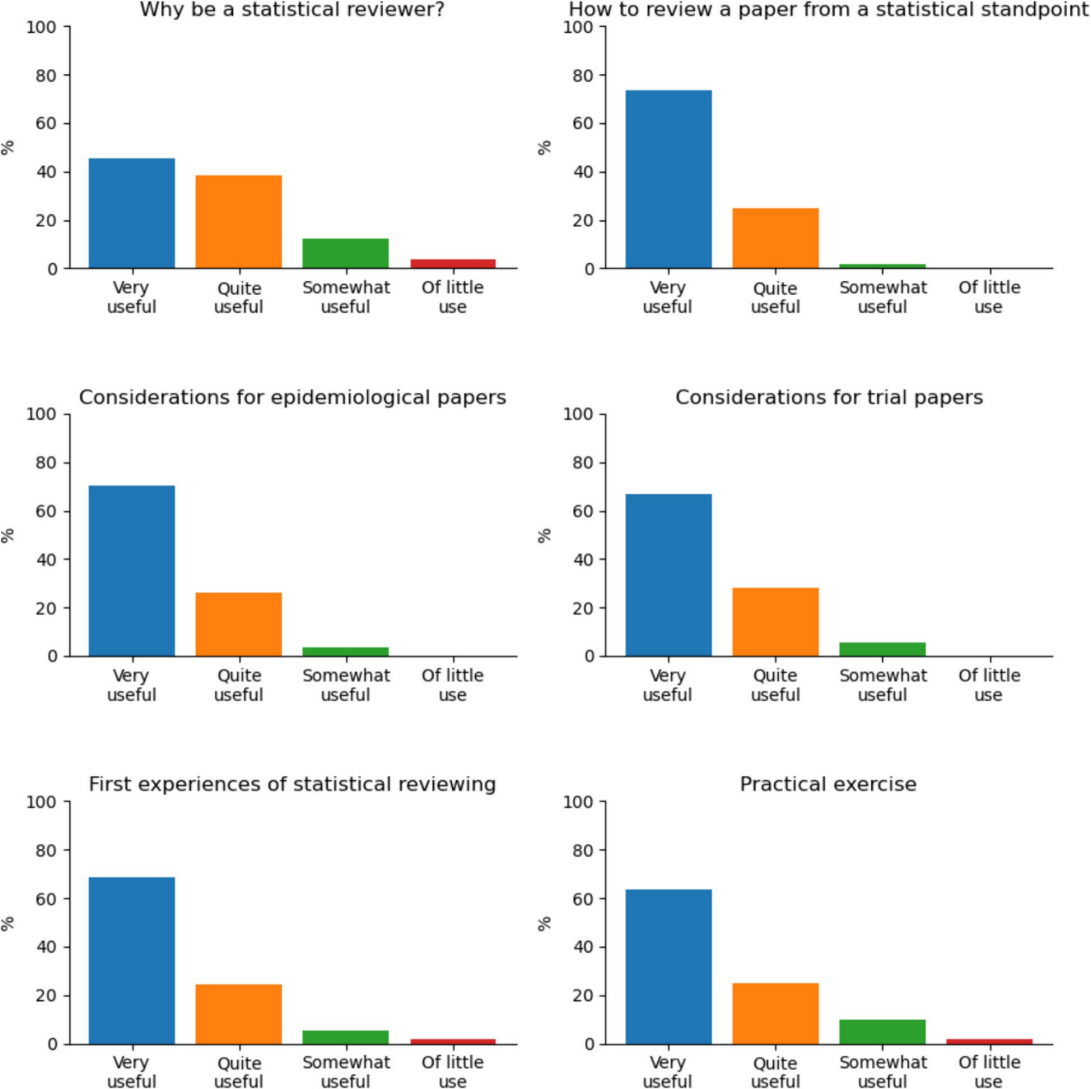
Workshop participants assesssment of the usefulness of each session

Figure 2 summarizes the participants’ views on the usefulness of each of the short talks and the practical session. The percentage of participants rating the talks as very useful ranged from 46 to 74% across the five talks, and the practical session was rated to be very useful by 63%.

Additional comments were provided by 32/58 (55%) responders. The value of the practical exercise was highlighted by many, including the option to select the study type of interest and receive pre-reading material. Many also commented on the usefulness of the talks, in particular the range of speakers. Other positive comments included the balance between presentation and participation, and the emphasis on considering constructive feedback alongside critical analysis. Suggestions for improvement included having smaller groups for the practical exercise, more prompting questions to guide discussion in the practical exercise, more guidance on writing a review in a constructive manner, providing model reviews for both papers, and deliberately selecting high quality papers so that discussion was not just focused on the flaws. Some requested the speaker slides (which are now available on the NIHR Statistics Group website along with a recording of the workshop; Webinar: A practical introduction to statistical reviewing for medical journals | NIHR Statistics Group), and several requested future similar events.

## Discussion

This article has summarised the content and learning from an online introductory workshop on statistical reviewing for medical journals that was organised by the Improving Statistical Literacy and Career Development working groups of the NIHR Statistics Group. The individual talks were based on the personal experiences and opinions of the speakers. The references are included in order to provide some support from the literature on the comments and experiences expressed.

The workshop was specifically designed to provide a practical introduction for early- or mid-career researchers with statistical training but little or no experience of statistical reviewing. The number of participants included for each of the practical sessions was determined by the number of attendees, their preference for reviewing a clinical trial or epidemiological paper and the number of facilitators available to help with the groups. Ideally smaller groups (5–10 participants) may be better to allow more time for discussion as with large groups it is possible that quieter participant’s may not have their say.

Key pieces of practical advice from the workshop topics are collected in Table 2. These are intended to provide a brief reference of tips and things to consider for those at the start of their journey as a statistical reviewer. While other sources of relevant advice exist (e.g. [26–28]) this article fills a gap by offering personal reflections from both experienced and inexperienced statistical reviewers, and addressing practical aspects of reviewing often not covered elsewhere, such as why review, how to review, and how to get started with reviewing. This article is not intended to provide an exhaustive checklist of statistical issues to consider when reviewing, or a collection of methodological comments frequently provided to authors (both of which are also excellent types of resources for a statistical reviewer to be able to consult) [26, 28, 29].

**Table 2:** A summary of practical advice from each presentation of the statistical reviewing webinar

Webinar session	Practical advice
Why be a statistical reviewer?	• If a statistical colleague is a reviewer for a medical journal, then ask about their experience such as how many reviews they have been asked to do and how long each one takes to complete. The time involved can then be gauged • Also enquire whether any journals are looking for new statistical reviewers. Statisticians are always in demand
General statistical reviewing	• The statistical reviewer should stay positive and focus on the overall message • It may help to imagine sitting down with the authors to go through the comments. Attention to detail is important but statistical reviewers should actively avoid excessive concern over minor issues or attempts to re-write or re-analyse • It is much easier to draft and revise the statistical review in a word processing package prior to submission onto a journal portal
Statistical reviewing of epidemiological papers	• Reporting guidelines and risk of bias tools are useful aids that can help reviewers ensure they are reviewing the relevant aspects of each study • The Strengthening the Reporting of Observational Studies in Epidemiology (STROBE) guidelines are a useful starting point [22]. • If reviewing a prediction model study, TRIPOD + AI [23] highlights the minimum required information that should be reported in these studies and PROBAST + AI [24] provides a risk of bias assessment • Details of validity assessment tools for evidence synthesis can be found at https://www.latitudes-network.org/about/
Statistical reviewing of clinical trial papers	• Statistical reviewers should utilise trial reporting guidelines like CONSORT 2025 [25] • More information on CONSORT and extensions to CONSORT reporting guidelines can be found at https://www.equator-network.org/ • General guidance on statistical assessment on medical papers are covered in the CHAMP reporting guidelines [26].
First time statistical reviewer	• Consider starting with a journal from a field in which you have some experience; this will help contextualise the statistics, making them easier to interpret and critique • If the opportunity is available, an induction with the editor will outline the process and the expectations for a statistical review, which can be very helpful and put you at ease • If there is an opportunity to perform a statistical review under the mentorship of a more experienced statistical reviewer this is likely to be helpful. Ask colleagues if there are opportunities to get involved or, if a journal invites you to perform a review, ask the editorial team if you can involve a colleague • As a statistician, with some background research you will have sufficient understanding to provide a good statistical review in most cases. But remember to draw the line in a sensible place: there are always things you could pick apart and critique in more detail • Keep in mind the main role, which is to ensure that the study design and methods are appropriate for the research question, they are executed and reported properly, and that the results justify the conclusions

Due to time constraints (this was a short online workshop of 2 h and thirty minutes duration), that aimed to give participants a flavour of statistical reviewing we were unable to collect information on whether there were differences in comments or topics focused on by participants with different levels of statistical reviewing experience. For pragmatic reasons, participants were simply asked to read the selected paper and focus on the methods and results section for the practical statistical reviewing component of the workshop. For those readers that are interested in more advanced training in statistical reviewing we are aware of initiatives such as those by Peerspectives (https://peerspectives.premier.charite.de/index.php/Peerspectives) that run courses that help train statistical reviewers.

It is important when providing a statistical review that the statistician adheres to a professional code of conduct and represent their capabilities and activities honestly and treat others with respect (e.g. https://www.amstat.org/your-career/ethical-guidelines-for-statistical-practice). The Committee on Publication Ethics (COPE) further emphasizes editorial strategies that ensure transparent and unbiased reviewer evaluations by trained professionals [30].

Scientific publishing, and hence science, relies on expert members of the academic and research community providing their expertise as peer reviewers. Statistical reviewing improves the quality and trustworthiness of research in medical journals and relies on those with appropriate statistical training and expertise providing their services as statistical reviewers. The authors of this article hope that it may serve to encourage more people to consider taking part in the important and rewarding activity of statistical reviewing for medical journals, and that it may be a source of helpful advice as they take their first steps in this role.

## Data Availability

All relevant data is presented in the manuscript.
